# The role of BMI across the life course in the relationship between age at menarche and diabetes, in a British Birth Cohort

**DOI:** 10.1111/j.1464-5491.2011.03489.x

**Published:** 2012-05

**Authors:** M B Pierce, D Kuh, R Hardy

**Affiliations:** MRC Unit for Lifelong Health and AgeingLondon, UK

**Keywords:** diabetes, longitudinal study, menarche, National Survey of Health and Development, women

## Abstract

**Aims:**

Previous research showing an inverse association between age of menarche and adult diabetes relied on recalled age at menarche and did not adjust for BMI across the life course. We investigated the relationship between age at menarche and diabetes, and whether childhood, adolescent or adult BMI attenuates this relationship.

**Methods:**

We used data from the Medical Research Council National Survey of Health and Development, a British birth cohort study of men and women born in 1946, with contemporaneous recording of the age of menarche, BMI at 2, 7, 15 and 20–53 years and diabetes status to 53 years.

**Results:**

A significant inverse relationship between age at menarche and diabetes [hazard ratio = 0.73 per year older age at menarche (95% CI 0.56–0.96), *P* = 0.02] was attenuated by adjustment for adult BMI [hazard ratio 0.85 (95% CI 0.65–1.10), *P* = 0.2]. The effect of age at menarche on Type 2 diabetes was very similar to that for all types of diabetes. Attenuation of the association between age at menarche and diabetes was also observed with BMI at 15 years, but less so with BMI measured earlier in childhood.

**Conclusions:**

Earlier age at menarche is associated with a higher risk of diabetes, and specifically Type 2 diabetes, in later life, which is most strongly attenuated by adolescent and adult adiposity. Early menarche may be clinically useful in identifying women who are at risk of later adiposity and so of developing Type 2 diabetes.

Diabet. Med. 29, 600–603 (2012)

## Introduction

As the prevalence of Type 2 diabetes is rising worldwide, there is increasing interest in risk factors for the disease, especially where there is a possibility of early intervention. Studies have suggested that earlier menarche is related to poorer glycaemic control in later life, but, to our knowledge, only two studies thus far have shown a relationship between early menarche and increased prevalence of diabetes [1, 2]. The associations were mediated by adult BMI, a well-established risk factor for Type 2 diabetes, which is also inversely related to age at menarche. These previous studies [1, 2] were subject to recall bias, as age at menarche, birthweight, body shape at 10 and weight at 18 years were reported in adulthood.

We use data from the Medical Research Council (MRC) National Survey of Health and Development (NSHD), a British birth cohort study of men and women born in 1946, in which contemporaneous BMI and the age of menarche are available for the majority of the girls. We investigate the relationship between age at menarche and diabetes, and assess whether childhood, adolescent or adult BMI attenuates this relationship.

## Patients and methods

The Medical Research Council National Survey of Health and Development is a birth cohort study of a stratified sample of 2547 women and 2815 men born in 1 week in 1946 [3]. There have been 21 follow-ups between birth and 53 years. Of the 3673 cohort members for whom contact was attempted at 53 years, 3035 (83%) provided information. The majority (*n* = 2989) were interviewed in their own homes by trained research nurses, with others completing a postal questionnaire (*n* = 46). Contact was not attempted for the 1689 individuals who had previously refused to take part (640), were living abroad (580) or had already died (469) [4]. The data collection received Multi-centre Research Ethics Committee (MREC) approval and informed consent was given by respondents to each set of questions and measures.

### Type of diabetes

From birth, all hospital attendances and reasons for attending were recorded. Doctor diagnoses of diabetes, dates of diagnoses and medications were reported at nurse interviews at 36, 43 and 53 years and on a postal questionnaire at 31 years. Relevant data of those with any report of diabetes or record of anti-diabetic medication were reviewed by a general practitioner with a special interest in diabetes (MBP). Individuals only ever treated with diet or oral hypoglycaemic agents, or who had insulin added more than 2 years after diagnosis, were classified as having Type 2 diabetes. All were 30 years or more at diagnosis.

Individuals who had taken insulin since time of diagnosis were classified as having Type 1 diabetes. All were under 29 years at diagnosis. Women with a diabetes diagnosis within 1 year prior to the birth of a child with no further record of diabetes treatment were classified as having gestational diabetes mellitus.

### Age at menarche

At 15 years, study members underwent a medical examination and interview by a school doctor. Age at menarche for girls was obtained from mothers’ reports at the examination. For 94 of the 188 girls who had not reached menarche by the time of the examination, age at menarche was obtained from cohort members’ reports when they were 48 years of age.

### Body size

At 36, 43 and 53 years, height and weight were measured at home visits by trained nurses according to standardized protocols. Height and weight were also measured at 2, 7 and 15 years, and self reported at 20 and 26 years.

BMI [weight (kg)/(height (m^2^)] was calculated at each age.

### Statistical methods

Cox’s proportional hazards models were used to estimate the association between age at menarche (in years) and incidence of diabetes. Follow-up was in months from birth until the diabetes diagnosis or the first of the following events: death, emigration or last completed questionnaire. Follow-up time was treated as censored if the event was other than diabetes diagnosis. In analyses with Type 2 diabetes as the outcome, similar models were used, except that follow-up of those with other forms of diabetes was censored at date of diagnosis. There were 1724 with a valid age at menarche.

BMI in adulthood was fitted in models as a time-dependent variable. Further analyses adjusted for BMI at 2, 7 and 15 years.

We tested whether the relationship between age at menarche and diabetes was linear by categorizing age at menarche into four groups and including those who had not reached menarche by the time of the 15-year examination in the oldest group.

The assumption of proportional hazards was checked by inspection of plots and found to be valid. Analyses were carried out in Stata 10 (StataCorp., College Station, TX, USA).

## Results

Thirty-eight women for whom we had age at menarche had reported diabetes by 53 years. Five had Type 1 diabetes, 26 had Type 2 diabetes, three had gestational diabetes and four were unclassifiable. The mean age at menarche was 13.2 years (range 8 years 6 months–19 years 6 months).

There was a significant inverse relationship between age at menarche and diabetes (Table 1). Analysis with age at menarche in categories suggested that this association was linear (results not shown). In the subgroup with measures of adult BMI, the effect of age at menarche was attenuated by adjustment for adult BMI and was no longer significant (Table 1).

**Table 1:** Hazard ratios for relationship between age at menarche in years and having any type of diabetes by age 53 years, with adjustments for BMI across the life course

		*n* (diabetes)	Hazard ratios (95% CI) per year later age of menarche	*P*-value
Full sample				
All diabetes	Unadjusted	1724 (38)	0.73 (0.56–0.96)	0.02
Sample with adult BMI				
All diabetes	Unadjusted	1632 (38)	0.73 (0.56–0.96)	0.02
	Adjusted for adult BMI	1632 (38)	0.85 (0.65–1.10)	0.2
Type 2 diabetes	Unadjusted	1632 (26)	0.72 (0.52–0.99)	0.05
	Adjusted for adult BMI	1632 (26)	0.86 (0.63–1.18)	0.4
Samples with adult BMI and each early life BMI				
All diabetes	Unadjusted	1275 (30)	0.74 (0.55–1.00)	0.05
	Adjusted for BMI at 2 years	1275 (30)	0.75 (0.56–1.01)	0.06
	Unadjusted	1481 (36)	0.75 (0.57–0.99)	0.04
	Adjusted for BMI at 7 years	1481 (36)	0.82 (0.63–1.09)	0.2
	Unadjusted	1525 (37)	0.77 (0.59–1.00)	0.05
	Adjusted for BMI at 15 years	1525 (37)	0.89 (0.67–1.19)	0.4
Subgroup with adult BMI and BMI at 7 and 15 years				
	Unadjusted	1381 (35)	0.79 (0.06–1.03)	0.09
	Adjusted for BMI at 7 years	1381 (35)	0.87 (0.66–1.14)	0.31
	Adjusted for BMI at 15 years	1381 (35)	0.92 (0.69–1.23)	0.59
	Adjusted for BMI at 7 and 15 years	1381 (35)	0.92 (0.69–1.23)	0.60
	Adjusted for adult BMI	1381 (35)	0.92 (0.70–1.21)	0.57
	Adjusted for adult BMI, BMI at 7 and 15 years	1381 (35)	0.94 (0.71–1.25)	0.69

The effect of age at menarche on Type 2 diabetes was very similar to that for all types of diabetes and was the same in the subgroup with adult BMI (Table 1). This association was similarly attenuated by adjustment for adult BMI (Table 1).

Little attenuation of the association between age at menarche and all diabetes by BMI at 2 years was observed. The effect on the association by adjustment for BMI at 7 years was greater, and the strongest attenuation was seen with BMI at 15 years (Table 1). This stronger attenuation after adjustment for BMI at 15 years is also observed in the subgroup, with measures of adult BMI and BMI at 7 and 15 years. Adjustment for adult BMI had the same effect on the association as adjustment for BMI at 15 years. After adjusting for BMI at age 15 years, adjustment for BMI at 7 years had no further effect on the association between age at menarche and diabetes.

## Discussion

In this prospective study we found that early menarche was associated with increased risk of diabetes, and specifically Type 2 diabetes, and that the relationship was most strongly attenuated by adolescent and adult BMI.

### Advantages and limitations

Unlike other studies [1, 2], age at menarche was assessed during adolescence in the current study. Age at menarche reported in adolescence and recalled in middle age has shown only moderate agreement [5]. For 94 of the 188 girls who had not reached menarche by 15 years, we used recalled age at menarche. Exclusion of a greater proportion of later-maturing girls because of missing age at menarche and use of recalled values may have resulted in some bias. However, similar, although weaker, relationships observed when treating menarche as a categorical variable with those who had not reached menarche by 15 years included in the latest maturing group, suggests little evidence of bias. The repeated measures of BMI throughout the life course are an advantage of our study, but there is limited power to assess how BMI at different ages attenuates the relationship between age at menarche and diabetes. This is also the first study to examine the effect of measured, as opposed to recalled [2] BMI in childhood and adolescence on the relationship between age of menarche and diabetes. Loss to follow-up is inevitable in a long-running cohort such as the National Survey of Health and Development [4]. The responding sample at 53 years was in most respects representative of a national sample of a similar age, whilst tending to under-represent the most disadvantaged groups [4]. Importantly, there was no difference in the distribution of age at menarche between those excluded from the analysis and those included. Lack of fasting blood glucose measurements made it possible that we missed a few unrecognized cases of diabetes.

### Comparisons with other studies

To our knowledge, only four previous studies have examined the relationship between age at menarche and diabetes. Lakshman *et al*. [1] found an inverse relationship between age at menarche and diabetes, but were unable to differentiate between Type 1 and Type 2 diabetes. He *et al*. [2] showed the same inverse relationship with Type 2 diabetes. A longitudinal study of 668 women found no association between age at menarche and adult-onset diabetes [6] and the Rancho Barnardo study [7] (*n* = 997) showed associations with blood glucose levels but not diabetes. The last two [6, 7] studies may have lacked statistical power because of their small size.

Adult BMI accounts for much of the association between age at menarche and diabetes. The association between earlier menarche and higher subsequent BMI is well established [8], but the nature of this association is much debated, as girls with early menarche are already heavier than their peers at menarche. We found less attenuation of the relationship between age at menarche and diabetes by childhood BMI at 7 years than at 15 years, although previous work on this cohort found that rapid childhood growth between 2 and 7 years was a risk factor for early menarche [9]. Consistent with our findings of less attenuation, previous studies have found that the relationship between age of menarche and BMI is not attenuated by childhood BMI (4–6 years) [10] and that childhood BMI (3–6 years) does not predict metabolic risk in late adolescence [11].

In conclusion, our study provides evidence that earlier age at menarche is associated with a higher risk of diabetes and, specifically Type 2 diabetes, in later life, which is most strongly largely attenuated by adolescent and adult adiposity. Early menarche may be clinically useful in identifying women who are at risk of later adiposity and so of developing Type 2 diabetes.

## Competing interests

Nothing to declare.
